# The Impact of Rhythmic Physical Activity on Mental Health and Quality of Life in Older Adults with and without Cognitive Impairment: A Systematic Review and Meta-Analysis

**DOI:** 10.3390/jcm12227084

**Published:** 2023-11-14

**Authors:** Marcelina Sánchez-Alcalá, Agustín Aibar-Almazán, Diego Fernando Afanador-Restrepo, María del Carmen Carcelén-Fraile, Alexander Achalandabaso-Ochoa, Yolanda Castellote-Caballero, Fidel Hita-Contreras

**Affiliations:** 1 Department of Health Sciences, Faculty of Health Sciences, University of Jaén, 23071 Jaén, Spainaaochoa@ujaen.es (A.A.-O.); mycastel@ujaen.es (Y.C.-C.);; 2 Faculty of Health Sciences and Sport, University Foundation of the Área Andina-Pereira, Pereira 660004, Colombia; dafanador4@areandina.edu.co; 3 Department of Education and Psychology, Faculty of Social Sciences, University of Atlántico Medio, 35017 Las Palmas de Gran Canaria, Spain

**Keywords:** rhythmic, dance, older adults, mental health, quality of life, systematic review, meta-analysis

## Abstract

(1) Background: Nowadays, it is essential to implement new non-pharmacological strategies, such as rhythmic physical activity, to improve mental health and quality of life in both individuals experiencing normal brain aging and those with cognitive impairment. Therefore, the objective of this study is to identify the effects of rhythmic physical activity interventions on mental health and quality of life in older adults, with or without mild cognitive impairment; (2) Methods: We conducted a systematic review with a meta-analysis, searching the Pubmed, Scopus, Web of Science, and Cochrane Plus databases using specific keywords. We selected studies that included rhythmic physical activity as the primary intervention for patients aged 65 and above, with or without cognitive impairment. We assessed the methodological quality of the articles using the PEDro scale; (3) Results: Out of 961 identified studies, we included 11 in this review, all of which employed rhythmic physical activity as an intervention. The selected studies consistently measured depression, anxiety, and quality of life; (4) Conclusions: This review demonstrates that rhythmic physical activity can effectively improve depression, anxiety, and quality of life in older adults, whether or not they have mild cognitive impairment. However, it is worth noting that while we have identified beneficial outcomes, the evidence supporting the use of rhythmic physical activity in enhancing depression, anxiety, and quality of life in older adults with or without mild cognitive impairment remains somewhat limited.

## 1. Introduction

Due to an increase in life expectancy, the global population of older individuals continues to grow [1]. This demographic shift has led to a higher prevalence of age-related conditions, including mild cognitive impairment (MCI) [2]. MCI represents an intermediate stage between normal cognitive aging and dementia. It is characterized by subtle cognitive changes that exceed age-related expectations but do not significantly disrupt daily functioning or meet the diagnostic criteria for dementia [3]. The recognition and research of MCI and dementia have seen a substantial growth in recent decades. According to estimates from the World Health Organization (WHO), approximately 16% of older adults are affected by MCI [4], while globally, around 50 million individuals are living with dementia [5]. Furthermore, projections suggest that, by 2030, the number of people with dementia will reach approximately 65.7 million [6], and this figure is expected to rise to 139 million by 2050 [5]. In recent years, there has been a growing interest in the study of MCI due to its significance in the early identification of potential dementia cases and the implementation of preventive interventions [7]. Recent studies [8,9] have provided insights on the nature of MCI, its prevalence, and its associated risk factors, highlighting MCI as an early indicator of neurodegenerative diseases like Alzheimer’s disease and similar disorders. Consequently, these studies underscore the importance of assessing and monitoring MCI to detect potential progression to dementia and enable early interventions. Both MCI and dementia, with their various stages and symptoms, profoundly affect cognition, thinking, and memory, resulting in reduced social functioning, challenging behaviors, and the expression of negative emotions. Among these symptoms, depression [10,11] and anxiety [12,13] are psychological aspects that warrant special attention.

The promotion of mental or psychological health is a fundamental element in the pursuit of healthy aging and in strategies to prevent mild cognitive impairment [14]. It is crucial to emphasize that mental health encompasses a state of psychological well-being and social cohesion, going beyond the mere absence of mental disorders [15] This entails promoting individual coping strategies, finding meaning in life, and experiencing personal growth as essential personal resources [16]. Research involving older adults has revealed a strong connection between negative emotional burdens and a higher incidence of brain disorders [15], alterations in brain functioning [17], an increased cognitive decline, and an elevated risk of developing Alzheimer’s disease [18]. Age-related health conditions are closely intertwined with psychological health, often leading to heightened negative emotions and, subsequently, a reduced perception of quality of life and overall well-being [19]. Conversely, psychological well-being has been linked to a lower prevalence of age-related health issues, including cardiovascular disease, cognitive impairment, and physical dysfunction [20]. Meta-analyses have provided support for the notion that a greater well-being is associated with a reduced risk of mortality [21,22]. Furthermore, among older populations, positive psychosocial factors such as social engagement, mindfulness, resilience to stress, and positive thinking patterns like optimism or a sense of purpose in life have been shown to correlate with improved brain structure and function, as well as an enhanced cognitive heal [23]. These positive aspects have also been demonstrated to decrease the risk of cognitive decline and the onset of dementia [20].

To effectively promote mental or psychological health, it is crucial that lifestyle-focused intervention strategies encompass emotional, psychological, physical, and social well-being [24]. These lifestyle practices, which aim to emulate multimodal enrichment in humans, should incorporate both physical and mental activities to foster an “embodied mind-in-motion” [25]. One eloquent example of such practices is dance or dance movement interventions (DMI), which seamlessly combine music (sensory), movement (physical), and mental stimulation within a socially enriching environment. Various programs fall under this umbrella, including traditional dance, dance aerobics, and dance/movement therapy. The latter is integrated into creative arts therapies and incorporates psychological elements and techniques such as creativity, emotional reflection, and psychosocial integration, all of which are intrinsic components of mental health and overall well-being [26]. As a result, these practices can be viewed as holistic tools that have the potential to promote healthy aging by enhancing physical, psychological, and social functioning in the older population [27]. Furthermore, evidence-based analyses have consistently demonstrated significant benefits in terms of cognitive health [28,29] and physical/physiological health [30,31] when compared to control groups among older adults. However, research examining the impact of IMD on psychological health and well-being, particularly in older adults without dementia, remains limited and inconclusive. Therefore, the goal of this study is to investigate the effects of a rhythmic physical activity intervention on mental health and quality of life in older adults, both with and without mild cognitive impairment.

## 2. Materials and Methods

This systematic review and meta-analysis adhere to the PRISMA 2020 guidelines [32], and the pre-specified protocol has been registered in PROSPERO (CRD42023455443).

### 2.1. Sources of Information

The literature review was conducted between July and August 2023 using the Pubmed, Scopus, Web of Science, and Cochrane Plus databases.

### 2.2. Search Strategy

Different keywords were used for the search, as well as the Boolean operators “AND” and “OR”, resulting in the following search string: (“rhythmic exercise” OR “rhythmic physical activity” OR “dance” OR “dancing” OR “dance therapy” OR “rhythmic task” OR “music-based” OR “rhythmic PA” OR “dance physical training” OR “square dance” OR “aerobic dance” OR “contemporary dance” OR “dance therapy” OR “dancing” OR “music exercise training” OR “dance-movement intervention”) AND (“mental health” OR “psychological well-being” OR “emotional well-being” OR “quality of life” OR “life quality” OR “health-related quality of life” OR “HRQOL”) AND (“older adults” OR “older women” OR “older men” OR “elderly” OR “seniors” OR “normal cognition” OR “without cognitive impairment” OR “mild cognitive impairment” OR “aging” OR “successful aging” OR “elderly people”).

### 2.3. Eligibility Criteria

The inclusion criteria for this review encompassed studies that were clinical trials or randomized controlled trials conducted with participants aged 65 years or older. These studies had to include at least one intervention group involving exercise or rhythmic physical activity and involve older adults, either with mild cognitive impairment, without cognitive impairment, or both. Additionally, the selected studies had to be conducted within the last 10 years. On the other hand, exclusion criteria consisted of studies lacking a non-intervention reference group, those not presenting comparative data between rhythmic physical training and the control group, or studies that did not provide information regarding mental health and quality of life. Publications such as books and papers, meta-analyses, systematic reviews, protocols, clinical trial registries, and articles that had not undergone peer review were also excluded. Lastly, studies not meeting the age criteria of participants (65 years or older) or those that were not peer-reviewed were not considered.

### 2.4. Study Selection Process

The search results were analyzed using the Rayyan QCRI tool, available at https://rayyan.qcri.org/welcome, accessed on 24 July 2023, which automatically removed duplicate articles. Two authors independently reviewed the titles and abstracts, evaluating their adherence to the inclusion criteria. Subsequently, they conducted a thorough reading of the selected articles. Any discrepancies were resolved through consensus, involving a third author when necessary.

### 2.5. Data Extraction

The primary focus of this review was to examine the impact of a rhythmic physical activity intervention on mental health, categorizing the effects based on the nature of the variables involved (e.g., depression, anxiety, psychological distress, stress, and quality of life). Each article was meticulously cataloged in a registry that included information such as its publication year, geographical origin, authorship, participant details (age, sample size, and distribution), intervention specifics (duration, intensity, and frequency), the assessment scales employed for each variable, timing of measurements, follow-up periods, and relevant statistical data.

### 2.6. Methodological Quality Assessment

The methodological quality of the included articles was assessed using the PEDro scale. Scores from the PEDro scale were obtained directly from the official source when available. In cases where this information was not available on the official website, two authors independently conducted the assessment. The PEDro scale consists of eleven criteria, with the first item excluded from the total score calculation due to its relation to external validity. As a result, only the scores for items 2 to 11 were summed. Methodological quality ratings were categorized as “poor” (0 to 3), “moderate” (4 to 5), “good” (6 to 8), and “excellent” (greater than 9).

### 2.7. Analytical Decisions of the Meta-Analysis

The findings are displayed through a forest plot graph, which includes information such as the lead author, publication date, the number of participants in each study, individual effects measured using the Hedge index (g), and the overall effect accompanied by a 95% confidence interval, along with the associated *p*-value. We conducted a sensitivity analysis that excluded studies with repeated individuals and unusual values, and then compared these results with those of the full meta-analysis.

For subgroup or stratified analyses, we grouped studies based on the type of scale used to measure each mental health variable. Within each group, separate meta-analyses were performed, allowing us to identify effect sizes and specific variability in each subgroup and providing a more detailed understanding of the results. Additionally, we used a meta-regression approach to assess how certain moderating variables, such as intervention frequency, duration, and volume, influenced the results.

Finally, we assessed the risk of publication bias using a funnel plot. This plot provides a visual representation of possible asymmetries that could indicate bias in the selection of studies for inclusion in the analysis.

## 3. Results

**Figure 1:**
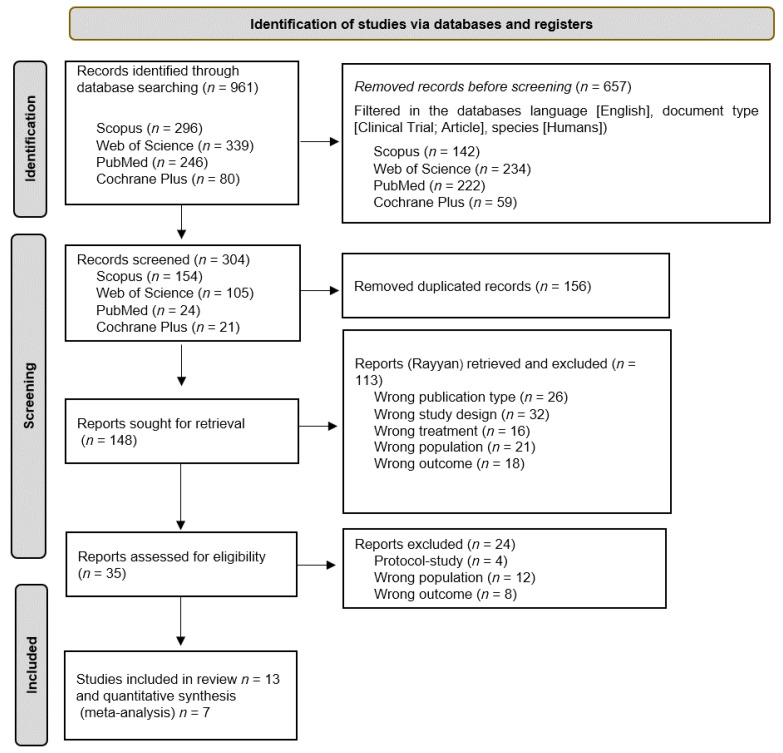
Flow diagram of the study selection process.

A comprehensive search was conducted across various databases, resulting in a total of 961 articles. Prior to the screening process, 156 duplicate articles were eliminated, leaving a total of 304 distinct articles. These articles were then screened based on their titles and abstracts, resulting in 148 articles that were further reviewed in full text. Out of these, 13 articles [33,34,35,36,37,38,39,40,41,42,43,44,45] were included in the systematic review, while 204 articles were excluded. The study selection process, following the PRISMA statement [32], is illustrated in Figure 1.

### 3.1. Methodology Quality

The methodological quality of the included studies was assessed using the PEDro scale. Scores for eight studies [33,35,36,38,40,42,43,44] were obtained from the PEDro web portal, while the remaining six [34,37,39,41,45] were assessed manually. Notably, none of the studies achieved the blinding of subjects or treatment providers. Additionally, it is important to highlight that three studies [37,39,45] were not randomized, and six articles did not allocate participants. Table 1 shows the PEDro scale evaluation scores.

### 3.2. Study Characteristics

All the studies included in this systematic review with meta-analysis were experimental studies, primarily conducted in China [33,34,36,39,44] and Malaysia [37,43], with additional studies from Greece [35], Spain [38], Canada [41], the United States [40,45], and Switzerland [42]. A total of 1025 older adults participated in these studies, with an average age of 73.07 ± 7.31 years. Participants were assigned to either control (*n* = 499) or intervention (*n* = 526) groups, where they received rhythmic physical activity-based treatments (Table 2).

Regarding the interventions, there was a significant heterogeneity in the types of rhythmic physical activities practiced. In terms of frequency, five studies [33,39,41,43,44] conducted interventions three times per week, seven studies [34,35,36,37,38,40,45] did so twice a week, and one study conducted sessions once a week. Seven of the studies [33,36,38,39,41,43,44] reported using moderate-intensity interventions, while the remaining four did not specify how intensity was controlled.

Furthermore, although only one study [33] included exclusively female participants, in all the studies, the female population was more representative than the male population.

### 3.3. Study Results

The primary focus of this systematic review with meta-analysis was mental health, specifically the presence of depression or anxiety within the study population. Regarding the depression assessment, seven studies [33,34,36,39,43,44,45] utilized The Geriatric Depression Scale (GDS-15), while four studies [37,38,40,42] employed the Hospital Anxiety and Depression Scale (HADS). One study [35] used the Beck Depression Inventory (BDI), while another study [41] used the State-Trait Anxiety Inventory (STAI) to assess anxiety.

Among the studies assessing depression, six reported statistically significant improvements favoring the rhythmic physical activity-based intervention [33,35,36,37,39,43]. Additionally, Cheung et al. [34], while not providing specific values for each measurement, observed a significant immediate improvement after the intervention (*p* < 0.001). However, these improvements diminished six weeks after the intervention’s conclusion (*p* = 0.052). On the other hand, three studies, utilizing the HADS [38,40,42], reported that the intervention did not yield statistically significant improvements in this variable (*p* > 0.05).

In terms of anxiety, two of the included studies [37,42] reported statistically significant improvements assessed using the Hospital Anxiety and Depression Scale (HADS). Additionally, Esmail et al.’s study noted that, while there was no improvement in the State-Trait Anxiety Inventory (STAI-Trait) (T0: 0.07 ± 1.09, T1: 0.16 ± 1.09; F: 0.220, *p* = 0.810), there was a significant improvement in the STAI-State (T0: 0.09 ± 0.86, T1: −0.11 ± 1.25; F: 5.010, *p* = 0.010). Conversely, two studies [38,40] did not observe any improvement in anxiety.

Regarding the secondary variable of this systematic review, which was quality of life, it was assessed in five studies. Two studies [33,39] used the Short-Form 12 health survey (SF-12), one [38] used the Short-Form 36 health survey (SF-36), and two more [37,40] used the Quality of Life-Alzheimer’s Disease (QoL-AD). Among these, Bisbe et al. reported no positive changes in the intervention group (Pre: 102.59 ± 5.47, Post: 99.76 ± 6.77, *p* = 0.088), while Chang et al. [33] observed an improvement only in the Physical Component Summary (Pre: 43.09 ± 6.49, Post: 44.69 ± 5.35, *p* = 0.011). The remaining three studies [37,39,40] reported statistically significant improvements (*p* < 0.05) in post-intervention quality of life.

### 3.4. Meta-Analysis

#### 3.4.1. Depression

**Figure 2:**
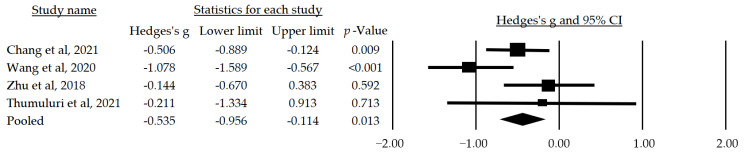
Forest plot of the mean effect size of physical activity-based interventions on depression as measured by The Geriatric Depression Scale [33,39,44,45].

In the meta-analysis, subgroup analyses were conducted using the assessment instrument and the presence of cognitive impairment as moderating variables. The first analyzed subgroup consisted of studies using the GDS-15 and included participants with cognitive alterations. In this subgroup, a statistically significant median effect size of *g* = −0.535 (CI: −0.956–−0.114, *p* = 0.013) favored interventions based on rhythmic physical activity (Figure 2).

**Figure 3:**
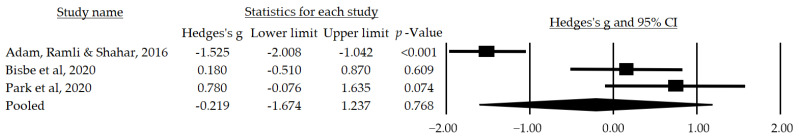
Forest plot of the mean effect size of physical activity-based interventions on depression as measured by the Hospital Anxiety and Depression Scale [37,38,40].

The second subgroup analysis comprised studies using the Hospital Anxiety and Depression Scale (HADS) and included participants with cognitive impairment. In this subgroup, a small mean effect size was observed, but it was not statistically significant (*g* = −0.219, CI: −1.674–1.237, *p* = 0.768) (Figure 3).

#### 3.4.2. Anxiety

**Figure 4:**
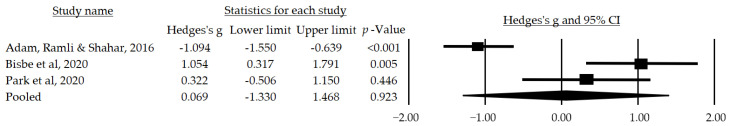
Forest plot of the mean effect size of physical activity-based interventions on anxiety as measured by the Hospital Anxiety and Depression Scale [37,38,40].

As for anxiety, a small mean effect size was observed, but it did not reach statistical significance (*g* = 0.069, CI: −1.330–1.488, *p* = 0.923) (Figure 4).

### 3.5. Risk of Bias

After the graphical analysis of each funnel plot resulting from the studies included in the different meta-analyses, it is evident that there is no apparent risk of publication bias, as indicated by the symmetrical distribution of the graph.

## 4. Discussion

This systematic review with meta-analysis encompassed 13 randomized controlled clinical trials [33,34,35,36,37,38,39,40,41,42,43,44,45], investigating the impact of rhythmic physical activity interventions on mental health and quality of life in older adults, with and without mild cognitive impairment. The results revealed a significant advantage favoring dance-based interventions over control groups, irrespective of the cognitive impairment status. These findings underscore the potential benefits of incorporating rhythmic physical activity into the lives of older adults for the enhancement of mental health and the overall quality of life.

In terms of methodological rigor, the majority of the included articles [33,34,35,36,38,40,41,42,44] demonstrated a good methodological quality, while four articles [37,39,43,45] presented a moderate level of methodological quality. Notably, none of the studies achieved an excellent quality rating. It is worth noting that the absence of patient or therapist masking and the inadequate allocation of assignments were common shortcomings across these studies, potentially influencing the observed results. Research has shown that the lack of patient or therapist masking and a suboptimal assignment distribution can contribute to increases of 13% and 7%, respectively, in the exaggeration of results [46].

Depression, a debilitating and highly prevalent mental disorder, profoundly affects the quality of life of those who suffer from it and its impact is not limited to the mental domain alone, but can also have significant physical and social consequences [47]. Our analysis supports the importance and feasibility of dance-supported interventions as an effective approach to address depression in individuals with or without mild cognitive impairment, as seven of the selected articles [33,36,37,39,43,44,45] showed a statistically significant improvement in depression as measured by the GDS-15 compared to control conditions in older adults with (*g* = −0.535, CI: −0.956–−0.114, *p* = 0.013) or without cognitive impairment. Based on our findings, the meta-analysis by Koch et al. [48] that focused on health-related psychological outcomes points out that dance therapy may have a positive impact on symptoms of depression. Similarly, the systematic review by Kiepe et al. [49], which addressed both physical and mental illnesses, also indicates that dance therapy could alleviate depressive psychological distress in patients. However, it is important to note that the review by Koch et al. [48] highlights a marked variability among the analyzed studies. In addition, both systematic reviews examined diverse illnesses, focusing on depression within the general population, without focusing on individuals with mild cognitive impairment.

Contrary to the results cited above, the present systematic review and meta-analysis presented three of the selected articles [38,40,42] that did not find statistically significant improvements in depression as measured by the HADS, which may be attributed to sample size, as these studies using the HADS had fewer participants. This may also be due to the control group employed, as the articles [33,35,36,37,39,43] that used the GDS-15 compared with control groups that continued their normal daily activities, whereas the articles [38,40,42] that used the HADS compared with other types of intervention.

On the other hand, anxiety is an emotional disorder that can manifest itself in a variety of symptoms such as excessive worry, agitation, and tension that can affect people’s ability to carry out their daily activities efficiently and enjoy everyday experiences [50]. In relation to the anxiety-related findings of this systematic review, four of the selected studies [37,38,40,42] measured this variable as assessed by the HADS (*g* = 0.069, CI: −1.330–1.488, *p* = 0.923) and the STAI-Trait and STAI-State [41]. Of these four studies that evaluated anxiety, two of them [37,42] showed statistically significant improvements. In agreement with our results, other studies have demonstrated the beneficial effects of a dance therapy intervention in the reduction of anxiety, but with other types of populations such as adolescents during the COVID-19 epidemic [51] and in internally displaced persons with depressive symptoms [52]. However, two other articles from the selected ones [38,40] suggested that, after the implementation of a dance-based intervention, no statistically significant differences were found in the decrease in anxiety in persons with and without cognitive impairment compared with a control group. Consistent with our results, the research synthesis by Bennett et al. [53] reflects the same outcome, although it incorporated a study that was not a randomized controlled trial. This fact underscores the need for additional evidence to corroborate the impact of dance-based interventions in reducing anxiety in individuals with or without mild cognitive impairment. Particularly, anxiety represents a risk factor [54,55], and dance-based interventions, as a modality of body psychological intervention, could address mental manifestations [56,57]. However, the expression of anxiety may differ from a typical early-onset anxiety disorder, and it is challenging to accurately identify and assess anxiety [58]. Therefore, further research is required to understand the effects of dance-based interventions in relation to anxiety.

Finally, quality of life is a multidimensional construct that encompasses physical, psychological, and social aspects of well-being, and reflects the subjective perception that people have of their own health status and how it is intertwined with their circumstances and environment [59]. In the present systematic review, five of the selected studies [33,37,38,39,40] measured the effects resulting from an exercise intervention on quality of life through a variety of measurement tools: SF-12, SF-36, and QoL-AD. Of these five studies, three [37,39,40] observed statistically significant improvements, and another study [33] only observed an improvement in the Physical Component Summary. Similar results are found in several systematic reviews such as that by Ma et al. [60] in which they explored the beneficial effects of rhythmic movement interventions on quality of life, but only in cognitively healthy older adults over the age of 60 years; despite these results, these authors recommended further studies with larger population samples, where the gender ratio is balanced, and follow-up is conducted over a prolonged period; the review by Fatkulina et al. [61] reported an improved quality of life in women diagnosed with breast cancer after dance/movement therapy; and the review by Lötzke et al. [62] revealed the positive effects on quality of life after an Argentine tango-based intervention in patients with Parkinson’s disease. In contrast to the previously mentioned results, this systematic review and meta-analysis found one study [38] that did not report positive changes in quality of life. Consistent with these results, a recent systematic review [63] found no significant improvements in quality of life in older adult patients with mild cognitive impairment, and the systematic review and meta-analysis by Carapellotti et al. [64] showed that dance had no beneficial effect on quality of life in Parkinson’s disease.

This review has several limitations that are worth noting. Firstly, it is important to acknowledge the absence of a blinding process. Neither the study participants nor the therapists responsible for administering the treatments were blinded, which could potentially introduce bias into the results. This lack of blinding may have influenced both the participants and therapists, impacting the objectivity of the results. Additionally, it is worth highlighting the geographical distribution of the included studies. The majority of the studies are from Asia, Europe, and America, while research conducted in Australia and Africa was not included. This geographical imbalance could potentially limit the generalizability of the results obtained in this review.

## 5. Conclusions

This systematic review, encompassing 11 randomized controlled trial studies, strongly suggests that rhythmic physical activity interventions, when compared to control groups, lead to significant improvements in depression, anxiety, and quality of life among older adults, regardless of whether they have mild cognitive impairment. These findings underline the potential of rhythmic physical activity in promoting healthy aging and the early intervention for age-related conditions and serving as a therapeutic approach for cognitive impairment. Health professionals and dance facilitators across various settings should consider the continued utilization of dance-based interventions for this demographic. Furthermore, there is a pressing need for further research with higher-quality randomized controlled experimental designs and larger sample sizes to build upon and strengthen the existing evidence base.

## Figures and Tables

**Table 1:** Methodological quality of the included articles.

Authorship	1	2	3	4	5	6	7	8	9	10	11	Total
Chang et al., 2021 [33]	1	1	0	1	0	0	1	1	0	1	1	6
Cheung et al., 2016 [34]	1	1	1	1	0	0	1	1	1	1	1	8
Lazarou et al., 2017 [35]	0	1	1	1	0	0	1	0	0	1	1	6
Ho et al., 2018 [36]	1	1	0	1	0	0	1	0	1	1	1	6
Adam, Ramli, and Shahar, 2016 [37]	1	0	0	1	0	0	0	1	0	1	1	4
Bisbe et al., 2020 [38]	1	1	1	1	0	0	1	1	1	1	1	8
Wang et al., 2020 [39]	1	0	0	1	0	0	1	1	0	1	1	5
Park et al., 2020 [40]	0	1	0	1	1	0	0	1	1	1	1	7
Esmail et al., 2019 [41]	1	1	1	1	0	0	1	1	0	1	1	7
Hars et al., 2013 [42]	1	1	0	1	0	0	1	0	1	1	1	6
Liao et al., 2018 [43]	1	1	0	0	0	0	1	1	0	1	1	5
Zhu et al., 2018 [44]	1	1	1	1	0	0	1	1	0	1	1	7
Thumuluri et al., 2021 [45]	1	0	0	1	0	0	0	1	1	1	1	5

Items: 1 = eligibility criteria; 2 = random allocation; 3 = concealed allocation; 4 = baseline comparability; 5 = blind subjects; 6 = blind therapists; 7 = blind assessors; 8 = adequate follow-up; 9 = intention-to-treat analysis; 10 = between-group comparisons; 11 = point estimates and variability; Y = Yes; N = No.

**Table 2:** Characteristics of the included studies.

Author and Year	Cognitive Impairment	Sex	Sample CG/IG	Control Group	Intervention Group			Measuring Instrument	Assessments	Values
					Age	Intervention	Intervention Parameters			
Chang et al., 2021 [33]	Mild mental disorder	F: 100%	47/62	Liberal daily lifestyle	76.56 ± 3.60	Square dance exercise	I: 100–140 beats per min F: 3 times/week #S: 54 sessions D: 30 min	GDS-15	T0: baseline T1: 9 weeks T2: 5 months	T0: 4.97 ± 1.41 T1: 4.55 ± 1.17 T2: 4.31 ± 1.14 *
Cheung et al., 2016 [34]	Moderate dementia	M: 25.9% F: 74.1%	53/58	Social activities	85.71 ± 6.68	Music-with-movement intervention	I: not reported F: 2 times/week #S: 12 sessions D: 30 min	GDS-15	T0: baseline T1: 6 weeks T2: 3 months	T0: 5.99 ± 3.57 T1: Not reported * T2: Not reported
Lazarou et al., 2017 [35]	Mild cognitive impairment	M: 21.7% F: 78.3%	63/66	Usual care	65.89 ± 10.76	International ballroom dancing	I: not reported F: 2 times/week #S: 80 sessions D: 60 min	BDI	T0: baseline T1: 10 months	BDI T0: 10.68 ± 5.89 T1: 8.27 ± 4.55 *
Ho et al., 2018 [36]	Mild dementia	M: 19% F: 81%	68/69	Waitlist control	79.4 ± 7.6	Dance movement therapy	I: moderate intensity F: 2 times/week #S: 24 sessions D: 60 min	GDS-15	T0: baseline T1: 3 months T2: 6 months T3: 12 months	GDS-15 T0: 0.8 ± 1.1 T1: 0.6 ± 0.9 * T2: 0.7 ± 1.0 T3: 0.7 ± 1.0
Adam, Ramli, and Shahar, 2016 [37]	Cognitively impaired elderly patients	M: 47.7% F: 52.3%	40/44	Relaxation exercises	70.3 ± 6.7	Combined dance and relaxation	I: not reported F: 2 times/week #S: 12 sessions D: 60 min	HADS	T0: baseline T1: 3 weeks T2: 6 weeks	HADS Depression T0: 7.6 ± 3.1 T1: 5.3 ± 3.0 T2: 3.7 ± 2.7 * Anxiety T0: 7.5 ± 3.5 T1: 6.1 ± 3.6 T2: 4.4 ± 2.7 *
Bisbe et al., 2020 [38]	Mild cognitive impairment	M: 52.9% F: 47.1%	14/17	Physical therapy	72.88 ± 5.60	Choreographed exercise	I: light-to-moderate F: 2 times/week #S: 24 sessions D: 60 min	HADS	T0: baseline T1: 3 months	HADS Depression T0: 6.59 ± 3.00 T1: 6.00 ± 3.43 Anxiety T0: 8.65 ± 4.18 T1: 8.82 ± 3.25
Wang et al., 2020 [39]	Mild cognitive impairment	M: 21.2% F: 78.8%	33/33	Usual lifestyle	81.06 ± 5.17	Chinese square dancing	I: moderate F: 3 times/week #S: 36 sessions D: 40 min	GDS-15	T0: baseline T1: 6 weeks T2: 3 months	T0: 4.88 ± 2.85 T1: 3.48 ± 2.32 T2: 2.61 ± 1.71 *
Park et al., 2020 [40]	Dementia	M: 58.1% F: 41.9%	11/10	Chair exercise	84.3 ± 7.7	Music intervention	I: not reported F: 2 times/week #S: 24 sessions D: 45 min	HADS	T0: baseline T1: 6 weeks T2: 3 months	HADS Depression T0: 9.40 ± 4.09 T1: 11.80 ± 4.10 T2: 12.30 ± 4.76 Anxiety T0: 5.40 ± 2.63 T1: 4.90 ± 3.54 T2: 7.89 ± 3.18
Esmail et al., 2019 [41]	Without cognitive impairment	M: 33.3% F: 66.7%	14/12	Usual care	68.08 ± 7.59	Dance/movement training	I: moderate F: 3 times/week #S: 36 sessions D: 60 min	STAI	T0: baseline T1: 3 months	STAI-State T0: 0.09 ± 0.86 T1: −0.11 ± 1.25 * STAI-Trait T0: 0.07 ± 1.09 T1: 0.16 ± 1.09
Hars et al., 2013 [42]	Without cognitive impairment	M: 3% F: 97%	68/66	Waitlist control	75 ± 8	Music-based multitask training	I: not reported F: 1 time/week #S: 25 sessions D: 60 min	HADS	T0: baseline T1: 6 months	HADS Depression T0: 6.59 ± 3.00 T1: 6.00 ± 3.43 Anxiety T0: 8.65 ± 4.18 T1: 8.82 ± 3.25 *
Liao et al., 2018 [43]	Without cognitive impairment	M: 38.3% F: 61.7%	52/55	Routine health education	71.79 ± 7.7	Combined music and Tai Chi	I: moderate F: 3 times/week #S: 36 sessions D: 50 min	GDS-15	T0: baseline T1: 1 month T2: 2 months T3: 3 months	T0: 15.8 ± 4.4 T1: 14.8 ± 4.4 T2: 14.2 ± 4.5 T3: 13.3 ± 4.3 *
Zhu et al., 2018 [44]	Mild cognitive impairment	M: 48.3% F: 51.7%	31/29	Usual care	70.3 ± 6.7	Aerobic dance routine	I: moderate F: 3 times/week #S: 36 sessions D: 35 min	GDS-15	T0: baseline T1: 3 months T2: 6 months	T0: 12.3 ± 7.2 T1: 10.4 ± 6.0 T2: 10.2 ± 7.0
Thumuluri et al., 2021 [45]	Early-stage dementia	M: 20% F: 80%	5/5	Usual care	74.15 ± 8.28	Improvisational movement	I: not reported F: 2 times/week #S: 16 sessions D: 60 min	GDS-15	T0: baseline T1: 2 months	T0: 1.2 ± 1.64 T1: 1.8 ± 1.48

I: intensity; F: frequency; #S: number of sessions; D: duration; CG: control group; IG: intervention group; T: assessment time; MH: maximum heart rate; GDS-15: The Geriatric Depression Scale; BDI: Beck Depression Inventory; HADS: Hospital Anxiety and Depression Scale; STAI: State-Trait Anxiety Inventory; and *: statistically significant within-group change.

## Data Availability

Not applicable.
